# The Control of Our Patients

**Published:** 1902-01

**Authors:** Henry A. Kelley

**Affiliations:** ’88, Portland, Me.


### THE CONTROL OF OUR PATIENTS.[1]

BY HENRY A. KELLEY, D.M.D., '88, PORTLAND, ME.

When I received an invitation to assist on Alumni Day, my thoughts naturally turned to the last time I appeared before you. This was in 1898, and I was one of six asked to discuss for five minutes, "The Present Status of Cataphoresis." Your committee in its invitation this year ask me to present something which will lead to a discussion, and for that reason I would like to make this paper a continuation of my former paper. For in the first place that will suggest to you cataphoresis, that in 1898 we all thought of so highly; and I would like to hear an expression from you as to how many are now using that method and with what results, and if you have discarded it, why you did so.

Another reason for making this a sequel to my former paper. As I was one of so many to consider the same subject, it pleased me to view the question in a way that I hoped would not be a repetition of the ideas of the other men. Therefore I took as my text the two questions, "How far had we progressed towards painless dentistry

---

[1] Read before the Harvard Dental Alumni Association, June 24, 1901.

when cataphoresis, as now practised, was presented to us?" and "How much farther has cataphoresis carried us?" I hardly flatter myself that those of you who heard that paper will remember what I said, but I assure you that it impressed me as a very incomplete paper. I can only say, in defence of it, that five minutes is a very short time in which to present any subject. So, with your permission, I will continue that paper to-day, taking up the side I then omitted. I may also say that a paper read by Professor Warner, of Springfield, before the Northeastern Dental Association last year, has been a great help to me in my endeavor to understand myself and express my thoughts to you; and I shall thus make use of some of his paper in my presentation of this subject. In my former article I expressed myself in this way. It has always seemed to me there must be two kinds of pain in a dental operation. One kind, that is the actual pain (the actual sensation), carried from the point of contact of the instrument to the brain, and another, perhaps we might say imaginary, that is manufactured in the brain. Any attempts to make dental operations painless must, in my mind, take this theory into consideration. And it was my idea that this first kind of pain must be relatively constant, and only to be controlled by the death or anæsthesis of the dental nerve; while the second, or mental pain, is very variable and potent, and can only be subdued by the way we can influence that mind to control itself.

While I felt the truthfulness of this position, it was my inability to express to you my thoughts, both from lack of time and of understanding myself, that made my little paper so unsatisfactory to me and, presumably, to you also. I used most of my time trying to tell you how to influence that troubled mind, and not in explaining the relations of mind to pain. Man enters life a stranger, but he is endowed with certain undeveloped senses by means of which external objects at once begin to make impressions upon his mind. Nature, whether animate or inanimate, is his complete master. At first he does not see things; he knows nothing about them. He only receives the stimuli which they furnish through the nervous system. But the inner self of this new creature answers these stimuli at first with primary sensations only, soon with ideas also. When first he opens his eyes he has no sight. He can neither see nor locate things. But he does not long remain blind. The power of perception is quickly created in his mind. He can soon see, hear, taste, smell, feel, and at last he conquers the world by perceiving it. It was formerly believed that the human mind passively received and recorded impressions like a mirror, but modern metaphysics teaches us, on the contrary, the mind is thoroughly active, transforming a physiological change into a psychical result,—*i.e.*, every nerve activity which is concerned in perception is a stimulus to which the mind responds with a distinctly different result,—a result which is peculiar to itself. Sensation really gives us little or no knowledge of the nature of the object. The sensations tell us rather how the object appears to us. It is hard for us to recognize the fact that we do not discover the true nature of things by our perceptions. Strictly speaking, what we call the properties and activities of things are only our sensations,—*i.e.*, psychical conditions arising from stimuli which proceed from the effect of external objects upon the nerves. When we speak of the sound of a bell, we commonly, but erroneously, consider that the sound belongs to the bell. As a matter of fact, the sensation we are thinking of is wholly a psychical condition. It is in our minds and belongs to us, not the bell. If this bell were rung where no life exists, it would produce no sound, if by sound we mean the sensations which we usually describe as the ringing of a bell. Suppose there were no other means of our acquiring a knowledge of this bell except by this one sensation, which we may now describe as a psychical condition created by the mind itself under the stimulus of a nerve change, which was occasioned by pulsations proceeding from the bell and falling upon the drum of the ear. Evidently this one sensation would give us very little of our present idea of a bell. It is thus easy to see that our idea of the bell is not a single mental image which the perceptions have engraved on the mind; but it is itself a creation of the active mind in all the various activities which have been stimulated by the several sensations occasioned by the contact which that bell has made with sense nerves through other forms of matter. The mind by its acting through one sense can create a very limited idea of the nature of things, and by the best exercise of all the senses it can still give us only an incomplete idea. This theory of ideas, as the product of the active mind, explains why it is no two persons have exactly the same ideas as the result of an observation of a given object. We do not perceive anything by a simple act of the mind in becoming conscious of mere excitations. As soon as there is the dawn of an idea, the foundation is laid for real perception. The sense powers build upon beginnings almost infinitesimally small. They gain in strength by exercise. The sense organs are furnished ready made, though undeveloped, for the purpose of giving the stimuli which are to furnish to the mind the evidences of the existence and nature of the external world. But the mind's resistance to these stimuli—*i.e.*, the sensations—is at first very feeble. The new-born infant may be supposed to stop with the mere sensation, because he is incapable of adding anything to it. Only in the most elementary sense can he be said to have any perceptions. He has a mind feebly active; he maintains a continual, though feeble, resistance against the stimuli that come through the sense organs and nerves, and somehow records these efforts or sensations. Soon he has recollections, comparisons, ideas, which he can bring to bear upon all new stimuli; then, and not till then, he begins to see, feel, hear, taste, and smell in a real sense.

It is in the recognition of this second act of perception, called apperception, that the new philosophy differs from the old. All our sensations, according to the modern theory, are largely the direct result of the activity of the mind in this secondary perception, assimilation, apperception. Simple, primary perceptions play an essential but relatively small part in this process. So soon as they arise in consciousness as the result of some stimuli from the special sense nerves, they are immediately joined to similar feelings or related ideas already in the mind. Thus held in consciousness for a time, they become cleared, more definitely connected with other related mental activities, and so assimilated or apperceived. THIS SECOND PSYCHICAL PROCESS ASSUMES AN IMPORTANCE IN ALL OUR SENSATIONS FAR BEYOND THE PRIMARY PERCEPTIONS, AND IT IS THIS FACT WHICH IS OF THE GREATEST SIGNIFICANCE IN SUGGESTING TRUE METHODS OF ACQUIRING KNOWLEDGE THROUGH THE SENSES. It leads to the inevitable conclusion that the character of our sensations and the kind of knowledge we acquire through them depend of necessity upon the character of the sensations previously apperceived and the knowledge already acquired. It would be difficult to trace with accuracy the character of the mental processes which go on in those more common and more limited perceptions, but it will not be doubted, I think, that the underlying principle is the same in all cases of sense activity; indeed, there is no lack of authority to sustain this view. All modern writers on this subject emphasize the idea of continuity in the capacity of sensation. This capacity does not spring afresh each time in the human being out of material incapable of sensation; but it is an acquired wealth of psychic power which has been created out of the proper use of an inherited endowment, without which there could be no sensation and no knowledge.

Every crude fact of nature that excites this power of sensation is transformed by it into a new fact whose character depends upon the immediate psychical use which the mind perceiving the fact is able to make of it. This is not saying that the mind creates the facts, that the world exists only in the living mind. Modern philosophy does not doubt the existence of the great universe, of facts apart from and independent from the mind, but when through sensation the mind perceives these crude facts, it can grasp them only as it can modify them to meet its own power of assimilation. Can you not understand from all this the necessity of taking the mind into account in your efforts to control the pain in your dental operations? I take it for granted—and I hope I am not in error—you have used all the mechanical and medicinal adjuncts to control the pain. And in doing this you have quite largely influenced the mind, because you cannot have done all this without impressing your patient with your kindness of heart, and if you have done that, you are far on the way to the control of your patient. I cannot here go over this ground, but in connection with the subject of mechanical and medicinal adjuncts I would recommend to your attention a paper entitled, "Our Mission: How shall We discharge It?" by B. Holly Smith, published in the April, 1900, *Dental Digest.*

To become personal, a long time ago, when I was receiving many patients for whom I had never operated, there was hardly a day passed but some one got out of my chair and remarked that never had he been hurt so little. Now I use all sorts of pain-producing instruments, and use them thoroughly; but somehow I do not make them hurt as some seem to do. And I honestly believe it is through their minds that I influence them. It is needless for me to enumerate all the things I do and you must do to gain this effect. My idea in bringing this subject before you is simply to impress you with the necessity of controlling the minds of your patients, and the truth of my statement that it can be done, and in conjunction with the mechanical and medicinal aids we have we can thus make our operations more easily endured by our patients. In closing, I would say I am not a Christian Scientist nor a hypnotist. I

believe there are such things as pain and disease in this world, and thus should take exceptions to the Christian Scientists. I believe, while hypnotism is so little understood by the masses, we, at least we younger men, had better not identify ourselves with it. In fact, after all, I think I am just a plain man with a kind heart. I recognize suffering, either of the body or mind, whenever I see it, and I maintain the necessity of controlling the one as well as the other if you are to have full control of your patients.

